# Genome editing in plants using the TnpB transposase system

**DOI:** 10.1007/s42994-024-00172-6

**Published:** 2024-06-08

**Authors:** Qi Li, Yongqiang Wang, Zhuoting Hou, Hang Zong, Xuping Wang, Yong E. Zhang, Haoyi Wang, Haitao Chen, Wen Wang, Kang Duan

**Affiliations:** 1 School of Ecology and Environment, Northwestern Polytechnical University, Xi’an, 710129 China; 2 Sanjie Institute of Forage, Yangling, 712100 China; 3 Key Laboratory of Zoological Systematics and Evolution, Institute of Zoology, Chinese Academy of Sciences, Beijing, 100101 China; 4 State Key Laboratory of Stem Cell and Reproductive Biology, Institute of Zoology, Chinese Academy of Sciences, Beijing, 100101 China

**Keywords:** ISAam1, ISDra2, ISYmu1, Plant genome editing, Rice, TnpB transposon endonuclease

## Abstract

The widely used clustered regularly interspaced short palindromic repeats (CRISPR)/CRISPR-associated nuclease (Cas) system is thought to have evolved from IS200/IS605 transposons. TnpB proteins, encoded by one type of IS200/IS605 transposon, are considered to be the evolutionary ancestors of Cas12 nucleases, which have been engineered to function as RNA-guided DNA endonucleases for genome editing in bacteria and human cells. TnpB nucleases, which are smaller than Cas nucleases, have been engineered for use in genome editing in animal systems, but the feasibility of this approach in plants remained unknown. Here, we obtained stably transformed genome-edited mutants in rice (*Oryza sativa*) by adapting three recently identified TnpB genome editing vectors, encoding distinct TnpB nucleases (ISAam1, ISDra2, and ISYmu1), for use in plants, demonstrating that the hypercompact TnpB proteins can effectively edit plant genomes. ISDra2 and ISYmu1 precisely edited their target sequences, with no off-target mutations detected, showing that TnpB transposon nucleases are suitable for development into a new genome editing tool for plants. Future modifications improving the genome-editing efficiency of the TnpB system will facilitate plant functional studies and breeding programs.

**Supplementary Information:**

The online version contains supplementary material available at 10.1007/s42994-024-00172-6.

Dear Editor,

In the past decade, gene editing technologies have enabled precise modifications of specific target genes (Knott and Doudna 2018; Wang et al. 2023). In particular, clustered regularly interspaced short palindromic repeats (CRISPR)/CRISPR-associated nuclease (Cas) (CRISPR/Cas) systems, including CRISPR/Cas9, CRISPR/Cas12, and CRISPR/Cas13, have been widely exploited for gene editing and have substantially promoted plant functional genomics research and crop breeding efforts (Zhou et al. 2023).

The class 2 CRISPR effectors Cas9 and Cas12 may have evolved, independently, from an ancestral TnpB-like nuclease, which is still commonly found in insertion sequences (ISs) of the IS200/IS605 and IS607 families of transposons (Altae-Tran et al. 2021; Kapitonov et al. 2016). Importantly, these transposases are only 400–500 amino acids (aa) in length, much smaller than Cas9 (1000–1500 aa) and Cas12a (1100–1300 aa) (Li et al. 2023). These small sizes may make these transposase systems feasible tools in cases where protein or nucleic acid size is a limiting factor. *Deinococcus radiodurans* ISDra2 TnpB (408 aa) (Karvelis et al. 2021; Meers et al. 2023) and OgeuIscB (496 aa), derived from the human gut metagenome (Schuler et al. 2022), have been engineered as RNA-guided DNA endonucleases for genome editing, along with retroelement-derived RNA (reRNA) and Obligate Mobile-Element-Guided Activity (referred to as OMEGA; ωRNA), as guide RNAs in bacteria and human cells, and their structures have been elucidated (Nakagawa et al. 2023; Schuler et al. 2022). Transposases are therefore interesting candidates as novel plant genome engineering tools.

A recent comprehensive analysis and characterization of the various TnpB proteins, encoded by members of the IS605 transposon family, established a method for large-scale mining of novel TnpB gene editors and yielded new miniaturized and highly active TnpB editors, such as ISAam1 (369 aa) and ISYmu1 (382 aa) (Xiang et al. 2023). The activities of the ISAam1 and ISYmu1 systems are similar to that of *Staphylococcus aureus* Cas9 and substantially higher than those of several reported Cas12f proteins and their variants, when expressed in human cells or *Escherichia coli*. However, the TnpB transposase system had not been used for plant gene editing. In this study, based on the characterization of TnpB transposases by Xiang et al. (2023) and using a well-established rice (*Oryza sativa*) transformation system, we have now tested the efficacy of the TnpB system for plant genome editing.

To investigate the ability of TnpB transposon endonucleases to edit target genes in stable transgenic plants, we tested three TnpB proteins: ISAam1, ISYmu1, and ISDra2 (408 aa) (Table S1). We placed the expression of the corresponding *TnpB* genes under the control of the rice *UBIQUITIN* promoter (*OsUBI*) (Fig. 1A). Considering that all three reRNA scaffolds in the TnpB systems start with a guanine (G), we chose the *OsU6a* promoter to transcribe the reRNA in rice, with the 20-bp target sequence at the 3′ end of the reRNA scaffold (Fig. 1A). ISAam1 recognizes a 5′-TTTAA transposon-associated motif (TAM), whereas ISYmu1 and ISDra2 both recognize a 5′-TTGAT TAM. Therefore, identical targets can be used for ISYmu1 and ISDra2. We chose *PHYTOENE DESATURASE* (*OsPDS*) as the first target gene in this study. OsPDS participates in chloroplast development, and its loss of function results in a visible phenotype of albino seedlings. For each TnpB, we designed two reRNAs targeting *OsPDS*: *OsPDS*-reRNA01 and *OsPDS*-reRNA02 for ISAam1, and *OsPDS*-reRNA03 and *OsPDS*-reRNA04 for ISYmu1 and ISDra2 (Fig. 1B; Table S2).

**Fig. 1:**
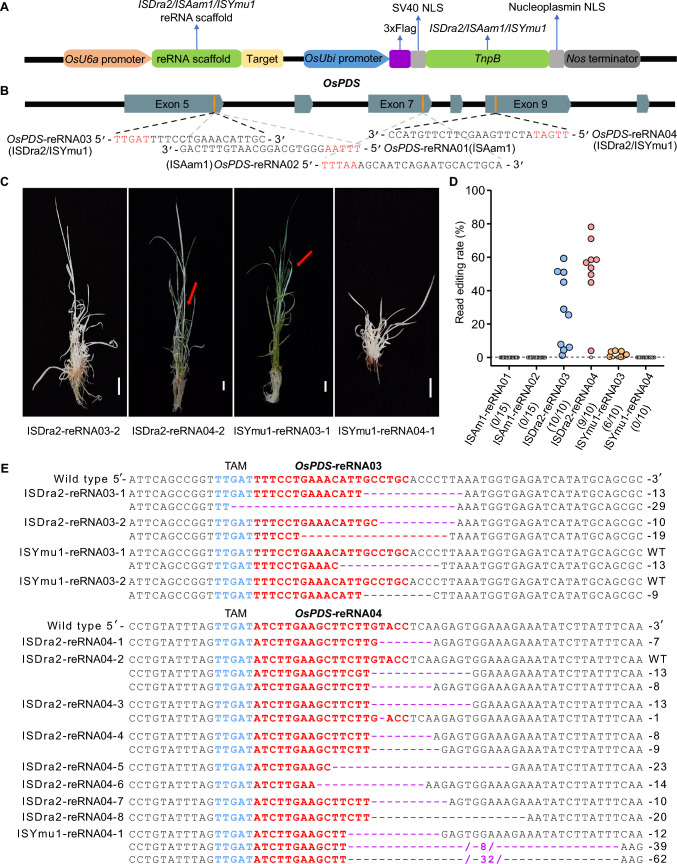
Plant genome editing using TnpB transposon nucleases. **A** The *TnpB* and *reRNA* expression cassettes were cloned into a genome editing vector for stable transformation of rice. *TnpB* was driven by the *OsUbi* promoter; *reRNA* (comprising the reRNA scaffold and target sequences) was driven by the *OsU6a* promoter. **B** The *OsPDS* locus with the target sites for each reRNA. *OsPDS*-reRNA01 and *OsPDS*-reRNA02 comprise a 5′-TTTAA transposon-associated motif (TAM) for ISAam1 recognition, whereas *OsPDS*-reRNA03 and *OsPDS*-reRNA04 include a 5′-TTGAT TAM for ISDra2 and ISYmu1 recognition. **C** Representative edited rice T_0_ mutant plants expressing *OsPDS*-reRNA03 and *OsPDS*-reRNA04 and showing an albino leaf phenotype. Red arrows indicate albino leaves. Scale bars, 1 cm. **D** NGS analysis showing the percentage of reads with mutations in T_0_ transgenic plants induced by each of the three TnpB systems. The dashed horizontal line represents a 0.1% editing frequency. Rice plants with editing frequencies < 0.1% were considered to lack gene editing events. **E** Multiple sequence alignment of the target sites for *OsPDS*-reRNA03 and *OsPDS*-reRNA04 in the wild type (Nipponbare) and T_0_ rice mutants edited by ISDra2 or ISYmu1

After stably transforming rice with each vector, we analyzed individual T_0_ transgenic plants originating from independent rice calli. Phenotypic analysis and Sanger sequencing results confirmed the successful editing of the two *OsPDS* target sites by the ISDra2 and ISYmu1 TnpB systems. For the ISDra2 system, three out of 53 transgenic plants obtained using the *OsPDS*-reRNA03 construct exhibited the expected albino leaf phenotype (including one with a partial albino leaf phenotype), and 18 out of 43 transgenic plants obtained with *OsPDS*-reRNA04 did so (Fig. 1C). The ISYmu1 system had a lower yield, with three out of 47 obtained using *OsPDS*-reRNA03 and only one out of 22 transgenic plants obtained using *OsPDS*-reRNA04 having albino leaves. Unfortunately, the ISAam1 TnpB system did not yield any plants with an albino leaf phenotype (out of 80 assessed) when either the *OsPDS*-reRNA01 or *OsPDS*-reRNA02 construct was used (Table S3).

To determine whether *OsPDS* had indeed been edited, we performed next-generation sequencing (NGS) validation. We sequenced PCR amplicons for each *OsPDS* target site from 15 transgenic plants for ISAam1 and ten plants each for ISDra2 and ISYmu1. The sequencing data indicated that none of the 30 transgenic plants tested that had been obtained using the ISAam1 system had been edited. By contrast, the ISDra2 system had produced gene editing at the two *OsPDS* target sites in all (10 out of 10) and most (9 out of 10) transgenic plants, respectively, although editing rates varied between plants. The ISYmu1 system produced gene editing in 5 out of 10 and 0 out of 10 transgenic plants at the two *OsPDS* target sites, respectively. Taking these results together with those for the plants sequenced by whole-genome sequencing for off-target assessment, the editing efficiencies at the two *OsPDS* target sites were zero (0/15 and 0/15) for the ISAam1 system, 100% (11/11) and 90.9% (10/11) for the ISDra2 system, and 90.9% (9/11) and 9.1% (1/11) for the ISYmu1 system, respectively (Fig. 1D, Table S4).

Further investigation revealed that relying on albino phenotypes to select plants missed many editing events in the transgenic plants obtained using the ISDra2 and ISYmu1 systems. Through immunoblotting using an anti-Flag antibody, we observed that TnpB proteins accumulated in many non-albino transgenic rice plants (Fig. S1A). Indeed, the protein levels for ISAam1-transgenic plants were comparable to those for ISDra2- and ISYmu1-transgenic plants. Cloning of PCR amplicons into a vector, followed by Sanger sequencing, showed that all *OsPDS* target sites in albino rice plants were indeed edited, with almost all mutant plants having deletion mutations at the target sites (Fig. 1E). To detect potential off-target effects induced by TnpB in vivo, we performed whole-genome sequencing of four *Ospds* mutants edited by ISDra2 and ISYmu1. We obtained a total of 57.6 Gb of clean data. We identified 12 potential off-target sites for *OsPDS*-reRNA03 and 21 for *OsPDS*-reRNA04, based on the presence of six or fewer mismatches between the genomic sequence and each sequence targeted by the reRNA. However, we did not detect off-target mutations in the whole-genome sequencing data from the four rice mutants (Table S5).

To further evaluate the gene editing efficiency of the three TnpB systems in rice, we chose two additional target genes (*YOUNG SEEDLING ALBINO* [*OsYSA*] and *CHITIN ELICITOR RECEPTOR KINASE 1* [*OsCERK1*]) and selected one target site for each TnpB system (Fig. S1B). We subjected rice callus to Agrobacterium-mediated transformation with each construct and collected the resulting transformed calli 15 days later for NGS of PCR amplicons containing the target site.

The ISAam1 system did not induce mutations in either *OsYSA* or *OsCERK1* in the 30 and 18 transformed calli examined, respectively. For *OsYSA*, we observed gene editing events in 25 out of 30 calli examined that had been produced using the ISDra2 system, with 0.2% reads showing mutations at the target site (averaged in all detected calli; hereafter abbreviated as “reads showing mutations”). The ISYmu1 system induced gene editing events in 28 out of 31 examined calli, resulting in 0.9% reads showing mutations. For *OsCERK1*, the ISDra2 system induced gene editing events in 26 out of 28 examined calli, with 0.7% reads showing mutations, and the ISYmu1 system induced gene editing events in all 20 examined calli, resulting in 1.3% reads showing mutations (Fig. S1C, Table S6). Thus, the ISDra2 and ISYmu1 TnpB systems can effectively edit plant genomes, although we did not detect any gene editing events when using the ISAam1 system.

Taken together, our results demonstrate for the first time the successful TnpB-mediated gene editing in plants and show that the ISDra2 and ISYmu1 nucleases possess considerable gene editing efficiency. Optimization of the TnpB editing system—in terms of promoter choice for expressing the editing components, target selection, codon optimization, and enzyme variant engineering—could further improve its editing efficiency. Further testing will also be required to assess its applicability to gene editing in dicots, since TnpB enzymes might differ in editing efficiency between dicots and monocots because of promoter and species specificity. Notably, TnpB nucleases recognize TA-rich TAMs, and thus TnpB systems should complement the Cas9 system, which recognizes the G-rich protospacer-adjacent motif NGG. Overall, our results have laid the groundwork for the development of additional plant genome editing tools.

## Materials and methods

### Plant materials and growth conditions

The rice (*Oryza sativa* L.) cultivar Nipponbare was used in this study. Plants were grown in damp soil (70% peat, 30% perlite mix) in growth chambers set at 28 °C and 60% relative humidity under long-day conditions (16-h light/8-h dark photoperiod) and 71.8 mmol/m^2^/s light intensity.

### Plasmid construction

The *OsU6a*-reRNA and *OsUBI* (*Oryza sativa UBIQUITIN*)-3xFlag promoter fragments were separately synthesized and cloned into the pUC57 vector at Tsingke (Xi’an, China). The reRNA sequences targeting the rice *PHYTOENE DESATURASE* (*OsPDS*) gene were designed and cloned into the *Sap*I or *Bsa*I sites of pUC57-*OsU6a*-reRNA using the Golden Gate assembly method (Tables S1 and S2). The resulting chimeric reRNA expression cassettes were then cloned between the *Sbf*I and *Kpn*I sites of pUC57-*OsUBI*-3xFlag. Full-length coding sequences of *TnpB* genes (*ISAam1*, *ISDra2*, and *ISYmu1*) codon-optimized for expression in human cells were individually PCR-amplified from the plasmids px330-*U6*-ISYmu1, px330-*U6*-ISAam1, and px330-*U6*-ISDra2 (Table S1) using primers listed in Table S7. These template plasmids were kindly provided by Professor Yong E. Zhang, one of the inventors of the TnpB system (Xiang et al. 2023). The resulting sequences were subcloned between the 3xFlag tag and the sequence encoding the nucleoplasmin nuclear localization signal (NLS) using the *Spe*I and *BamH*I sites in pUC57-*OsUBI*-3xFlag. Finally, the complete TnpB system cassette, between the *Sbf*I and *BamH*I sites, was cloned into the pCAMBIA1300 vector using T4 DNA ligase. The resulting constructs were used to stably transform rice plants, as detailed below. The vectors for the three TnpB systems targeting *OsYSA* or *OsCERK1* were constructed by replacing the *OsPDS* reRNAs using the restriction enzymes *Sbf*I and *Kpn*I (Fig. S1B, Table S2).

### Generation of stable transgenic rice plants

The pCAMBIA1300 vectors containing each TnpB and reRNA expression cassette were individually transformed into Agrobacterium (*Agrobacterium tumefaciens*) strain EHA105 using the freeze–thaw method. Agrobacterium-mediated transformation of Nipponbare rice callus was conducted as described (Duan et al. 2012).

### Mutation screening by sequencing

Genomic DNA was extracted from T_0_ rice plants using the cetyltrimethylammonium bromide (CTAB) method (Allen et al. 2006). Transgenic plants were screened by PCR for the presence of the hygromycin resistance gene. Subsequently, *OsPDS* was PCR-amplified from transgenic plants carrying the hygromycin resistance gene, and the resulting amplicons were sequenced by Sanger sequencing at Tsingke (Xi’an, China). The *OsPDS* amplicons from representative T_0_ plants with albino phenotypes were cloned into the pEasy Blunt vector (Transgen Biotech, China) to enable a detailed evaluation of the mutated sequences. To accurately assess the mutations in *OsPDS*, 15 ISAam1, 10 ISDra2, and 10 ISYmu1 transgenic plants were chosen. The *OsPDS* gene was amplified from these plants, and then NGS of the amplicons was performed at Biorun (Wuhan, China). To exclude sequencing errors, only read mutation rates above 0.1% were considered. Similarly, genomic DNA was extracted from 15-day-old transgenic rice callus using the CTAB method after *Agrobacterium tumefaciens*-mediated transformation with transgenes targeting Os*YSA* or Os*CERK1*. The Os*YSA* and Os*CERK1* targeted fragments were PCR-amplified and subjected to NGS to assess read mutation rates.

### Immunoblot analysis

Total proteins were extracted (50 mM Tris–HCl, 150 mM NaCl, 1 mM EDTA, 4 M urea, 1 mM PMSF, 0.1% Nonidet P-40) from transgenic rice leaves (1-cm leaf in length from 1-month-old transgenic rice plants in growth chambers) and equal volumes of extract were analyzed by immunoblotting (Tian et al. 2023). A mouse anti-DDDDK-Tag mAb (1:10,000; AE005, ABclonal) antibody was used to detect Flag-tagged TnpB, and an HRP*-conjugated Goat Anti Mouse IgG(H + L) (1:5000; RS0001, Immunoway) secondary antibody was used for protein signal detection.

### Detection of off-target mutations

To detect off-target mutations, four *Ospds*-type rice mutants (ISDra2-reRNA03-2, ISDra2-reRNA04-2, ISYmu1-reRNA03-1, and ISYmu1-reRNA04-1) were chosen for whole-genome sequencing. The TruSeq Library Construction Kit (Illumina, San Diego, CA, USA) or the MGIEasy FS DNA Prep Kit (BGI, China) was used to build the library, which was then sequenced using the Illumina DNBSEQ-T7 sequencing platform (HAORUI GENOMICS, China). Fastp (v 0.20.1) (Chen et al. 2018) was used to evaluate raw data quality and exclude low-quality reads as well as reads shorter than 15 nucleotides. The cleaned reads were mapped to the Nipponbare rice reference genome with BWA-MEM (v. 0.7.17) (Li and Durbin 2010). SeqKit (v 2.2.0) (Shen et al. 2016) was used to predict potential off-target sites in the rice genome (parameters: -i -m 7 -j 1) with fewer than 6 mismatches in the 20-bp reRNA sequence. These putative off-target mutations in the whole-genome sequencing reads were manually examined to validate whether they were indeed mutations induced by the TnpB system. As previously reported (Peterson et al. 2016), single-nucleotide variations were excluded; only insertion/deletion (InDel) events at or near the 3′ position relative to the target sequence were considered to be TnpB-induced off-target mutations.

### Supplementary Information

Below is the link to the electronic supplementary material.

Supplementary file1 (PDF 631 KB)

Supplementary file2 (XLSX 30 KB)

## Data Availability

The raw data from whole-genome sequencing have been deposited at the National Genomics Data Center (NGDC) under accession number PRJCA021730.
