# Supplementary material for: Genome editing in plants using the TnpB transposase system
Source: aBIOTECH. 2024 Jun 8;5(2):225–30. doi: 10.1007/s42994-024-00172-6 (PMC11224200; doi:10.1007/s42994-024-00172-6)
Supplement: Supplementary file 1 — Supplementary file1 (PDF 631 KB) [file 42994_2024_172_MOESM1_ESM.pdf]

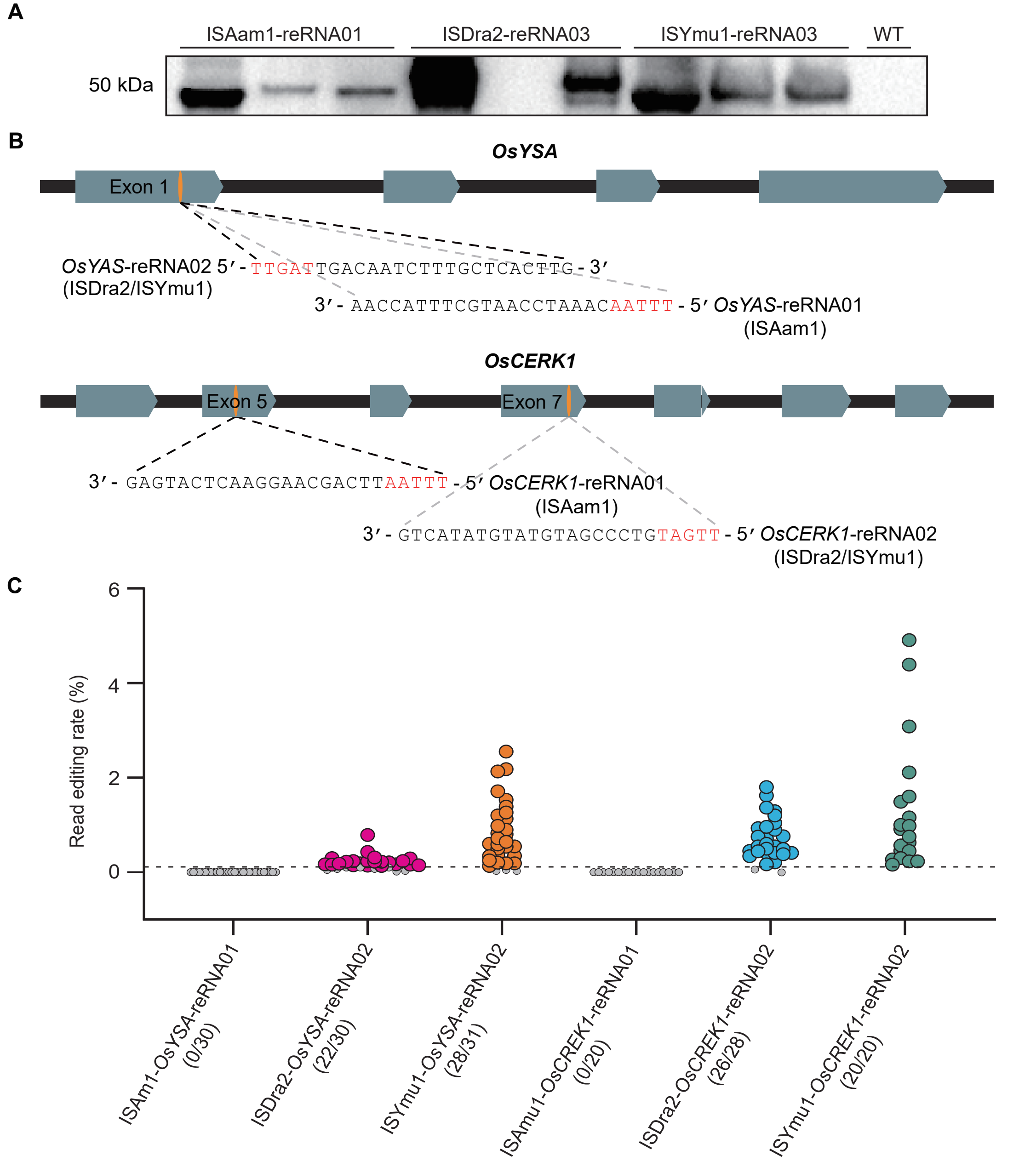

Fig. S1 TnpB protein expression in transgenic rice plants and assessment of the editing efficiency of TnpB systems in rice calli. **A** Immunoblot analysis of TnpB proteins in transgenic rice plants harboring edited versions of *OsPDS*, revealing the levels of the ISAam1, ISDra2, and ISYmu1 proteins in transgenic plants targeting *OsPDS* using the three TnpB systems. Three individual plants were analyzed per system. The sizes of the ISAam1, ISDra2, and ISYmu1 proteins with a Flag tag are 49.7 kDa, 52.8 kDa, and 50.9 kDa, respectively. **B** Partial gene structures of *OsYSA* and *OsCERK1*, with sequences targeted for mutation below each structure. The targets *OsYSA*-reRNA01 and *OsCERK1*-reRNA01 with 5'-TTTAA TAM for the ISAam1 system, and the targets *OsYSA*-reRNA02 and *OsCERK1*-reRNA02 with 5'-TTGAT TAM for both the ISDra2 and ISYmu1 systems. Red type represents the TAMs of the three TnpB systems. **C** NGS read editing rate of transgenic calli at 15 d after Agrobacterium infiltration with the indicated constructs using the three TnpB systems. Dashed line represents a read editing rate of 0.1%; read editing rates of <0.1% were considered not to reflect any gene editing events.
